# Mammary Tumors Induce Central Pro-inflammatory Cytokine Expression, but Not Behavioral Deficits in Balb/C Mice

**DOI:** 10.1038/s41598-017-07596-9

**Published:** 2017-08-15

**Authors:** William H. Walker II, Jeremy C. Borniger, Surbhi, Abigail A. Zalenski, Stevie L. Muscarella, Julie A. Fitzgerald, Ning Zhang, Monica M. Gaudier-Diaz, A. Courtney DeVries

**Affiliations:** 1 Department of Neuroscience, The Ohio State University Wexner Medical Center, 460W 12th Ave., Columbus, OH 43210 USA; 2 Neuroscience Research Institute, The Ohio State University Wexner Medical Center, 460W 12th Ave., Columbus, OH 43210 USA; 3 Behavioral Neuroendocrinology Group, The Ohio State University Wexner Medical Center, 460W 12th Ave., Columbus, OH 43210 USA

## Abstract

Breast cancer survivors are more likely to develop mood disorders and cognitive deficits than women in the general population. Previous studies suggest that peripheral tumors elicit central pro-inflammatory cytokine production, in turn leading to depression and cognitive deficits. In the current study, two cohorts of female Balb/C mice received bilateral orthotopic injections of syngeneic 67NR, 4T07, or 4T1cells (1 × 10^5^ cells per injection) to induce mammary tumors. Approximately three weeks later, learned fear (via fear conditioning) or depressive-like behavior (via tail suspension and forced swim test) was assessed. Proinflammatory cytokine levels were increased in the serum (IL-1β, TNFα, IFNγ) and livers (IL-1β, IL-6, TNFα) of mice with 4T07 or 4T1 tumors compared to 67NR tumors and the vehicle control. IL-1β was increased in both the hippocampus and cortex of mice injected with 4T07 or 4T1 cell lines relative to the other treatment groups. However, mammary tumors had no effect on hippocampal doublecortin + and did not alter depressive-like behavior or learned fear. These data demonstrate that similarly sized tumors can produce differential immune responses and that tumor-induced central pro-inflammatory cytokine production can exist in the absence of depressive-like behavior or cognitive deficits.

## Introduction

Breast cancer is one of the most common types of cancer among women in the United States. According to the National Cancer Institute and American Cancer Society, there are currently more than 3 million breast cancer survivors in the United States, and approximately 252,000 women will be diagnosed with breast cancer in 2017. Unfortunately, these women are 25–30% more likely to be diagnosed with a mood disorder relative to the general population; indeed, some estimates suggesting that as many as 50% of breast cancer survivors could develop a comorbid mood disorder^1, 2^. Although many breast cancer survivors will go on to develop a comorbid mood disorder, the most common of these are depression and anxiety, symptoms also may manifest as cognitive disturbances^1^. These findings are of major concern, because comorbid depression, along with cognitive disturbances, are associated with reduced quality of life, reduced treatment compliance, increased cancer progression, and reduced survival rates^3–9^.

The cause of cancer-related affective disorders and cognitive deficits has not been conclusively established. However, the inflammatory and neurodegenerative hypothesis of depression has been proposed to explain these deficits^10^. Several studies indicate that peripheral tumors, induced via carcinogens or metastatic cell lines, result in central pro-inflammatory cytokine production and the development of depressive-like behavior^11–14^. Along with activating the peripheral immune system, mammary tumor cells can secrete proinflammatory cytokines including interleukin 6 (IL-6) and interleukin 1 beta (IL-1β)^11, 15^; these signals are then likely transmitted to the brain via humoral or vagal signaling^16–19^. Within the brain, these proinflammatory cytokines are hypothesized to activate microglia, which then propagate this signal, in turn contributing to the induction of depressive-like behavior and cognitive deficits^18^.

Whereas potential mechanisms underlying the transmission of peripheral cytokine signals to the brain are well understood, the mechanisms through which central pro-inflammatory cytokine production can induce depressive-like behavior and cognitive deficits remain speculative. There are proposals regarding how this phenomenon occurs. Current hypotheses include (1) degradation of tryptophan along the kynurenine pathway through actions on indoleamine 2–3-dioxygenase (IDO), (2) decreased neurogenesis, or (3) increased neurodegeneration through actions on neurotrophins, particularly brain-derived neurotrophic factor (BDNF)^10, 18^.

Thus, affective deficits have been documented in tumor-bearing rodents, but whether these behavioral deficits are related to central pro-inflammatory cytokine production and altered neurogenesis remains to be determined. Thus, the current study sought to compare the effects of three types of orthotopic mammary tumors on peripheral and central inflammation, as well as hippocampal neurogenesis (DCX + cells), affective behaviors, and cognitive function. We generated tumors using multiple cell lines (67NR, 4T07, and 4T1) obtained from Barbara Ann Karmanos Cancer Institute (Detroit, MI); these cell lines are distinct isolated subpopulations from a single spontaneous Balb/cfC3H mammary tumor, and differ in their ability to induce secondary metastasis^20^.

## Results

### Cohort 1

#### Body Mass Changes and Tumor Cytokine Production are Cell Line Specific

**Figure 1:**
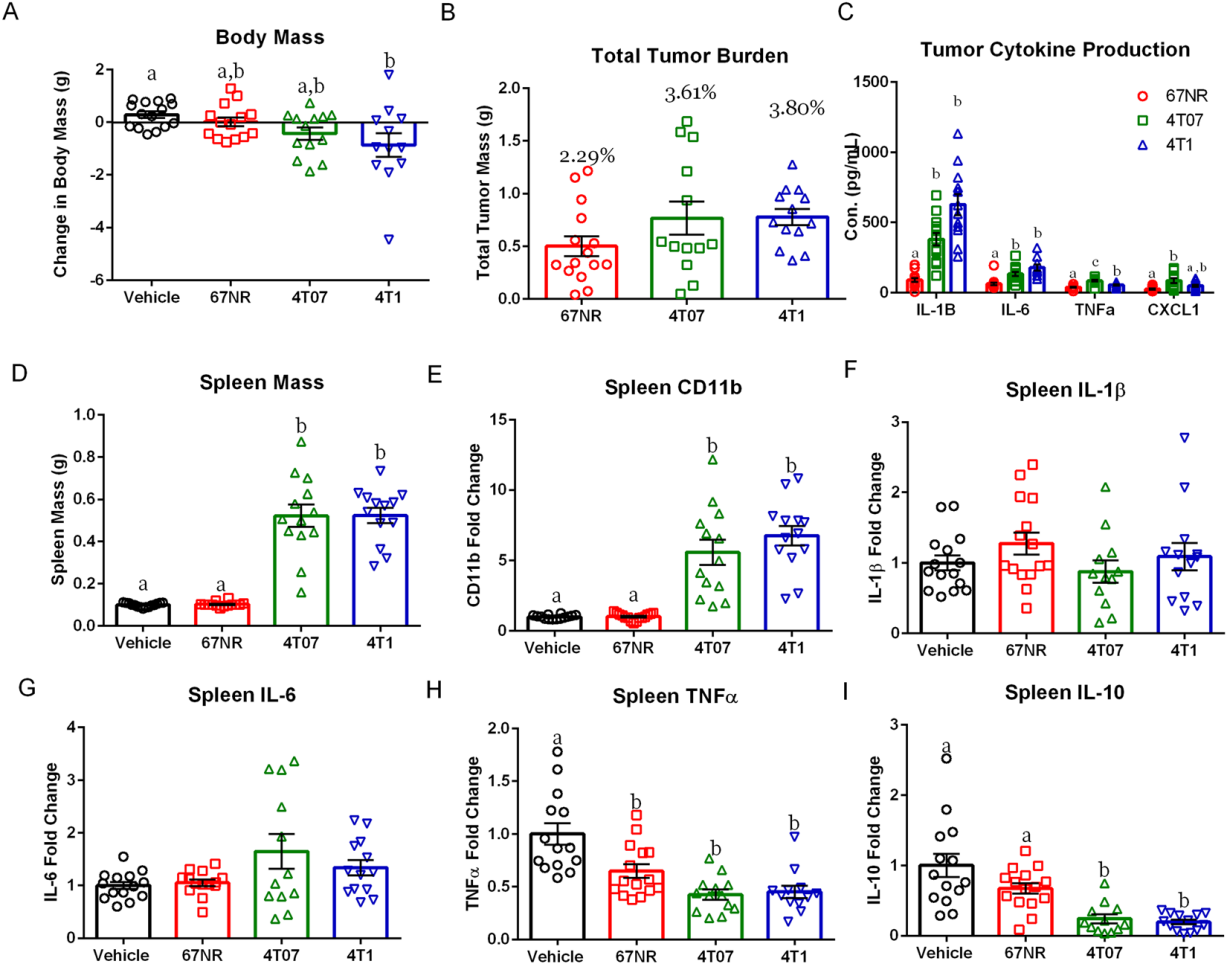
The Effects of Mammary Tumors on Body Mass and the Peripheral Cytokine Response Are Cell Line Specific. (**A**) Change in body mass between date of tumor inoculation (DOI) and date of euthanasia (DOE) accounting for tumor mass (TTB) for vehicle and each cell line ((DOE-DOI)-TTB); only mice with 4T1 tumors had significant body mass loss relative to the vehicle controls. (**B**) Total tumor burden in grams, and as a percentage of body mass, did not differ between cell lines. (**C**) Protein cytokine concentrations of IL-1β, IL-6, and TNF-α were elevated in 4T07 and 4T1 tumors relative to 67NR tumors. CXCL1 was elevated in 4T07 tumors relative to 67NR and 4T1 tumors. (**D**) Spleen mass was elevated among mice with 4T07 and 4T1 tumors relative to 67NR tumors and vehicle treated mice. (**E**–**I**) Spleen mRNA expression expressed as fold change from vehicles. (**E**) Macrophage marker CD11b was elevated in 4T07 and 4T1 tumors relative to vehicles and 67NR tumors. (**F** and **G**) Spleen IL-1β and IL-6 mRNA did not differ between groups. (**H**) TNFα mRNA expression was significantly reduced in all cell lines relative to vehicles.(**I**) IL-10 expression was significantly reduced in 4T07 and 4T1 tumors relative to 67NR tumors and vehicles. The measures and tissues were collected 20 days after tumor cell or vehicle inoculation (n = 12–15/group). The data are presented as mean + SEM. Graph bars that do not share a letter are statistically significant different at p < 0.05.

Cachexia is a common side effect of late stage cancer. There was a significant effect of treatment on body mass (F_3,50_ = 3.92, p < 0.05) measured on the final day of the experiment; mice injected with 4T1 cells had reduced body mass compared to vehicles (Fig. 1A, p < 0.05, Tukey’s multiple comparisons). The experimental groups injected with either 67NR or 4T07 cells had mean body weights that were intermediate between the vehicle treated group and the 4TI treated group, but not significantly different from either (p > 0.05). To rule out a potential confound associated with tumor size, tumor masses were recorded but did not differ among groups (Fig. 1B, F_2,38_ = 2.04, p > 0.05). There were no group differences in tumor protein concentrations of IFN-γ, IL-2, IL-4, IL-5, IL-10, and IL-12p70 (Fig. 1C, p > 0.05 data not shown). However, there was a significant effect of tumor cell type on IL-1β (Fig. 1C, H = 29.44, p < 0.0001), IL-6 (Fig. 1C, F_2,37_ = 13.68, p < 0.0001), TNFα (Fig. 1C, F_2,38_ = 26.73, p < 0.0001), and CXCL1 (Fig. 1C, H = 10.86, p < 0.005). Specifically, tumors generated with 4T07 or 4T1 cells had significantly greater IL-1β (p < 0.05, Dunn’s multiple comparisons) and IL-6 (p < 0.05, Dunn’s multiple comparisons) concentrations than tumors generated with 67NR cells. Tumors generated with 4T07 cells had significantly more TNFα (p < 0.05, Tukey’s multiple comparisons) than tumors generated with either 4T1 or 67NR cells. TNFα production also was significantly elevated in 4T1 tumors relative to 67NR tumors (p < 0.05, Tukey’s multiple comparisons). CXCL1 concentration was significantly elevated in 4T07 tumors when compared to 67NR tumors (p < 0.05, Dunn’s multiple comparisons), and 4T1 tumors had an intermediate concentration of CXCL1, with no significant differences relative to 67NR or 4T07 tumors (p > 0.05, Dunn’s multiple comparisons).

#### Mammary Tumors Alter Spleen Mass and Cytokine Production

There was a significant effect of treatment on spleen mass (Fig. 1D, H = 40.50, p < 0.001). Mice injected with either 4T07 or 4T1 cells displayed a significant increase in spleen mass when compared to either the vehicle or 67NR treated groups (p < 0.05 in all cases, Dunn’s multiple comparisons test). There was a concomitant effect of treatment on CD11b mRNA levels (Fig. 1E, H = 40.76, p < 0.001), with 4T07 or 4T1 treated groups exhibiting significantly greater CD11b mRNA expression in the spleen when compare to either the 67NR or vehicle treated groups (Fig. 1E, p < 0.05 in all cases, Dunn’s multiple comparisons). TNFα mRNA expression was reduced among all tumor bearing groups compared to vehicle treated animals (Fig. 1H, F_3,50_ = 13.32, p < 0.001, p < 0.05 in all cases, Tukey’s multiple comparisons). Spleen IL-10 mRNA expression also was reduced among 4T07 or 4T1 tumors when compared to either 67NR or vehicle treated groups (Fig. 1I, H = 29.54, p < 0.05 in all cases, Dunn’s multiple comparisons). However, there were no significant differences among groups in spleen mRNA levels of IL-1β (Fig. 1F, F_3,51_ = 1.185, p > 0.05) or IL-6 (Fig. 1G, H = 2.735, p > 0.05)

#### Mammary Tumors Increase Serum Cytokine Concentrations

**Figure 2:**
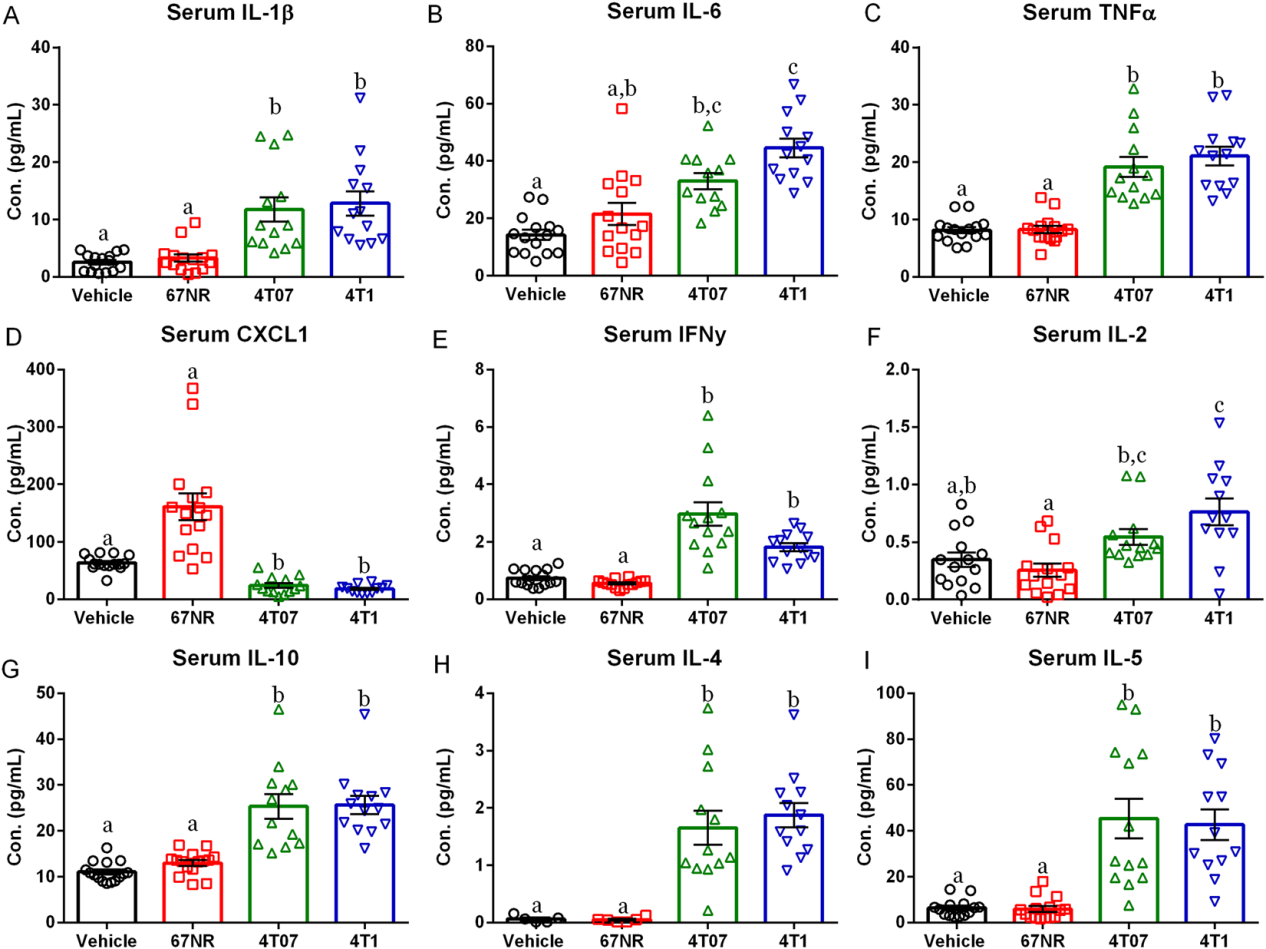
4T07 and 4T1 Tumors Increase Serum Cytokine Concentrations. (**A**–**I**) Serum protein cytokine concentrations of IL-1β, IL-6, TNFα, CXCL1, IFNγ, IL-2, IL-10, IL-4, and IL-5 expressed in both vehicle and treatment groups. (**A**) Serum IL-1β was significantly increased in 4T07 and 4T1 tumors relative to 67NR tumors and vehicles. (**B**). Serum IL-6 was increased in 4T07 and 4T1 tumors relative to vehicles. (**C**) TNFα concentrations were significantly increased in 4T07 and 4T1 tumors relative to 67NR tumors and vehicles. (**D**) Serum CXCL1 was significantly reduced in 4T07 and 4T1 tumors relative to 67NR tumors and vehicles. (**E**) IFNγ concentrations were significantly increased in 4T07 and 4T1 tumors relative to 67NR tumors and vehicles. (**F**) Serum IL-2 was increased in 4T07 and 4T1 tumors relative to 67NR tumors. (**G**–**I**) Serum IL-10, IL-4, and IL-5 was significantly increased in 4T07 and 4T1 tumors relative to 67NR tumors and vehicles. The measures and tissues were collected 20 days after tumor cell or vehicle inoculation (n = 12–15/group). The data are presented as mean + SEM. Graph bars that do not share a letter are statistically significant different at p < 0.05.

There were significant effects of treatment on concentrations of IL-1β (Fig. 2A, H = 35.91,p < 0.0001), IL-6 (Fig. 2B, F_3,50_ = 19.90,p < 0.0001), TNFα (Fig. 2C, H = 40.65,p < 0.0001), IFNγ (Fig. 2E, H = 41.74,p < 0.0001), IL-2 (Fig. 2F, F_3,49_ = 8.266,p < 0.0001), IL-10 (Fig. 2G, H = 40.03,p < 0.0001), IL-4 (Fig. 2H, H = 22.52,p < 0.0001), and IL-5 (Fig. 2I, H = 37.13,p < 0.0001), along with a decrease in CXCL1 (Fig. 2D, H = 43.75,p < 0.0001). IL-12p70 failed to reach a detectable range in the serum. Specifically, mice treated with either 4T07 or 4T1 cells had significantly higher concentrations of IL-1β, TNFα, IFNγ, IL-10, IL-4, and IL-5, along with a significant reduction in CXCL1, when compared to either the vehicle or 67NR treated group (Fig. 2A–I excl. B and F, p < 0.05 in all cases, Dunn’s multiple comparisons). Serum IL-6 and IL-2 was increased in mice receiving 4T1 cell injections when compared to either vehicle or 67NR treated mice (Fig. 2B and F, p < 0.05, Tukey’s multiple comparisons). 4T07 treated mice had significantly elevated serum IL-6 only when compared to vehicle mice and a significant increase in serum IL-2 only when compared to 67NR treated mice (2B and F, p < 0.05, Tukey’s multiple comparisons). At this time point (Day 20 post-tumor inoculation), the vehicle and 67NR groups did not differ significantly in any cytokine concentration. There were no correlations between tumor cytokine concentrations and serum cytokine concentrations for any cytokine measured (data not shown, p > 0.05 in all cases, Pearson’s correlation coefficient).

#### Mammary Tumors Increase Liver Inflammation

**Figure 3:**
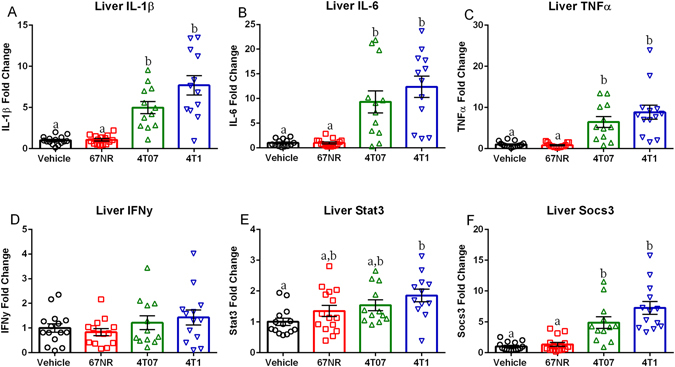
4T07 and 4T1 Tumors Increase Liver Inflammation and Downstream IL-6 Signaling. (**A**–**D**) Liver cytokine mRNA expression of IL-1β, IL-6, TNFα, and IFNγ expressed as fold change from vehicle.(**A**–**C**) Liver IL-1β, IL-6, and TNFα were significantly increased in 4T07 and 4T1 tumors relative to 67NR tumors and vehicles. (**D**) There were no significant differences in liver IFNγ. (**E–F**) mRNA levels of downstream IL-6 signaling molecules, STAT3 and SOCS3 expressed as fold change from vehicle. (**E**) STAT3 mRNA expression was increased in 4T1 tumors relative to vehicles. (**F**) Liver Socs3 mRNA was significantly higher in 4T07 and 4T1 tumors relative to 67NR tumors and vehicles. The measures and tissues were collected 20 days after tumor cell or vehicle inoculation (n = 12–15/group). The data are presented as mean + SEM. Graph bars that do not share a letter are statistically significant different at p < 0.05.

Mice with either 4T07 or 4T1 tumors had increased liver mRNA production of IL-1β (Fig. 3A, H = 34.10, p < 0.001), IL-6 (Fig. 3B, H = 29.12, p < 0.001), and TNFα (Fig. 3C, H = 35.31, p < 0.001) compared to either the vehicle or 67NR tumor-bearing mice (Fig. 3A–C, p < 0.05 in all cases, Dunn’s multiple comparisons). However, no group differences in IFNγ mRNA were detected (Fig. 3D, F_3,50_ = 1.297, p > 0.05). Likewise, there were group differences in STAT3 (Fig. 3E, F_3,49_ = 4.261, p < 0.01) and Socs3 (Fig. 3F, H = 35.52, p < 0.001) expression; specifically, mice with 4T1 tumors had increased STAT3 mRNA levels when compared to vehicle treated mice (Fig. 3E, p < 0.05, Tukey’s multiple comparisons), while mice with 4T07 or 67NR tumors displayed an intermediate phenotype which was not significantly different from any other group (Fig. 3E, p > 0.05, Tukey’s multiple comparisons). Liver Socs3 mRNA expression was increased in both 4T07 and 4T1 compared to the vehicle and 67NR treated mice (Fig. 3F and I, p < 0.05 in all cases, Dunn’s multiple comparisons).

#### Mammary Tumors Result in Central Pro-inflammatory Cytokine Production

**Figure 4:**
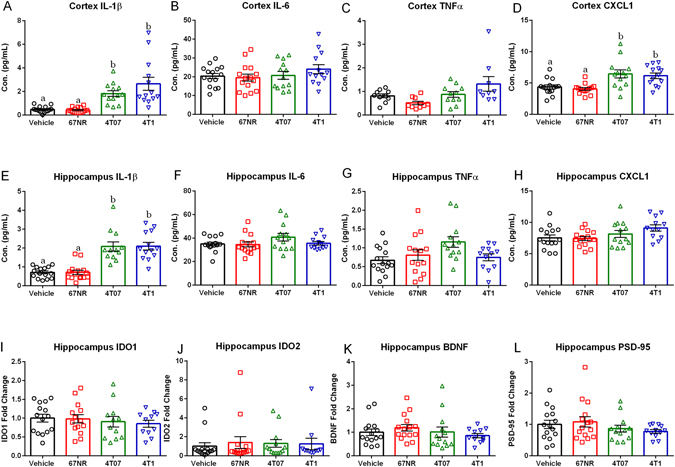
4T07 and 4T1 Cell Lines Induce Central Pro-inflammatory Cytokine Production. (**A**–**D**) Cortex protein cytokine concentrations of IL-1β, IL-6, TNFα, and CXCL1 expressed in both vehicle and treatment groups. (**A**) Cortex IL-1β was significantly increased in 4T07 and 4T1 tumors relative to 67NR tumors and vehicles. (**B**) There were no significant differences in cortex IL-6 (**C**) Cortex TNFα was significantly increased in 4T1 tumors relative to 67NR tumors. (**D**) Cortex CXCL1 was significantly increased in 4T07 and 4T1 tumors relative to 67nR tumors and vehicles. (**E**–**H**) Hippocampus protein cytokine concentrations of IL-1β, IL-6, TNFα, and CXCL1 expressed in both vehicle and treatment groups. (**E**) Hippocampus IL-1β was significantly increased in 4T07 and 4T1 tumors relative to 67NR tumors and vehicles. (**F**) There were no significant differences in hippocampal IL-6. (**G**) Hippocampal TNFα was significantly increased in 4T07 tumors relative to vehicles. (**H**) There were no significant differences in hippocampal CXCL1. (**I**–**L**) mRNA levels expressed as fold change from vehicles for IDO1 (enzyme implicated in etiology of depression), IDO2 (enzyme implicated in etiology of depression), BNDF (neurotrophin), and PSD-95 (marker of postsynaptic densities); There were no significant differences in mRNA expression of IDO1, IDO2, BDNF, or PSD-95. The measures and tissues were collected 20 days after tumor cell or vehicle inoculation (n = 12–15/group). The data are presented as mean + SEM. Graph bars that do not share a letter are statistically significant different at p < 0.05.

Mice with 4T07 or 4T1 tumors had significantly elevated cortical concentrations of IL-1β (Fig. 4A, H = 37.07, p < 0.0001) and CXCL1 (Fig. 4D, H = 19.26, p < 0.001), along with increased hippocampal concentrations of IL-1β (Fig. 4E, F_3,51_ = 24.04, p < 0.0001) compared to either the vehicle or 67NRtumor groups (Fig. 4A,D,E, p < 0.05, Tukey’s multiple comparisons). These increases in IL-1β were further confirmed in both the hippocampus (data not shown, H = 34.29, p < 0.001) and cortex (data not shown, H = 34.84, p < 0.001) via qPCR, which showed elevations that were identical to the protein data. However no group differences existed in cortical IL-6 (Fig. 4B, F_3,52_ = 1.037, p > 0.005), TNFα (Fig. 4C, F_3,36_ = 3.964, p > 0.005), IFNу (data not shown, H = 5.565, p > 0.005), IL-4 (data not shown, F_3,47_ = 0.1261, p > 0.005), IL-5 (data not shown, F_3,50_ = 1.885, p > 0.005), IL-10 (data not shown, F_3,48_ = 0.042, p > 0.005), or hippocampal IL-6 (Fig. 4F, F_3,52_ = 1.727, p > 0.005), TNFα (Fig. 4G, F_3,50_ = 3.197, p > 0.005), CXCL1 (Fig. 4H, F_3,51_ = 2.673, p > 0.005), IFNγ (data not shown, F_3,50_ = 2.037, p > 0.005), IL-2 (data not shown, F_3,51_ = 1.212, p > 0.005), IL-4 (data not shown, F_3,52_ = 1.171, p > 0.005), IL-5 (data not shown, F_3,52_ = 2.111, p > 0.005), IL-10 (data not shown, F_3,52_ = 0.6636, p > 0.005), or IL-12p70 (data not shown, F_3,46_ = 0.4249, p > 0.005). Concentrations of two cytokines IL-2 and IL-12p70 failed to reach the detectable range in the cortex. Further, no significant differences existed in IDO1 (Fig. 4I, F_3,48_ = 0.3495, p > 0.05), IDO2 (Fig. 4J, F_3,48_ = 0.1111, p > 0.05) BDNF (Fig. 4K, F_3,50_ = 0.7519, p > 0.05), PSD-95 (Fig. 4L, F_3,51_ = 1.095, p > 0.05), or iNOS (data not shown, F_3,47_ = 1.827, p > 0.05) as measured by qPCR. Also, there was no significant correlation in 4T07 or 4T1 treated mice between serum IL-1β or TNFα and IL-1β or TNFα concentrations in either the hippocampus or cortex (data not shown, p > 0.05, Pearson’s correlation coefficient).

#### Mammary Tumors Do Not Alter Locomotor Activity or Depressive-like Behavior

**Figure 5:**
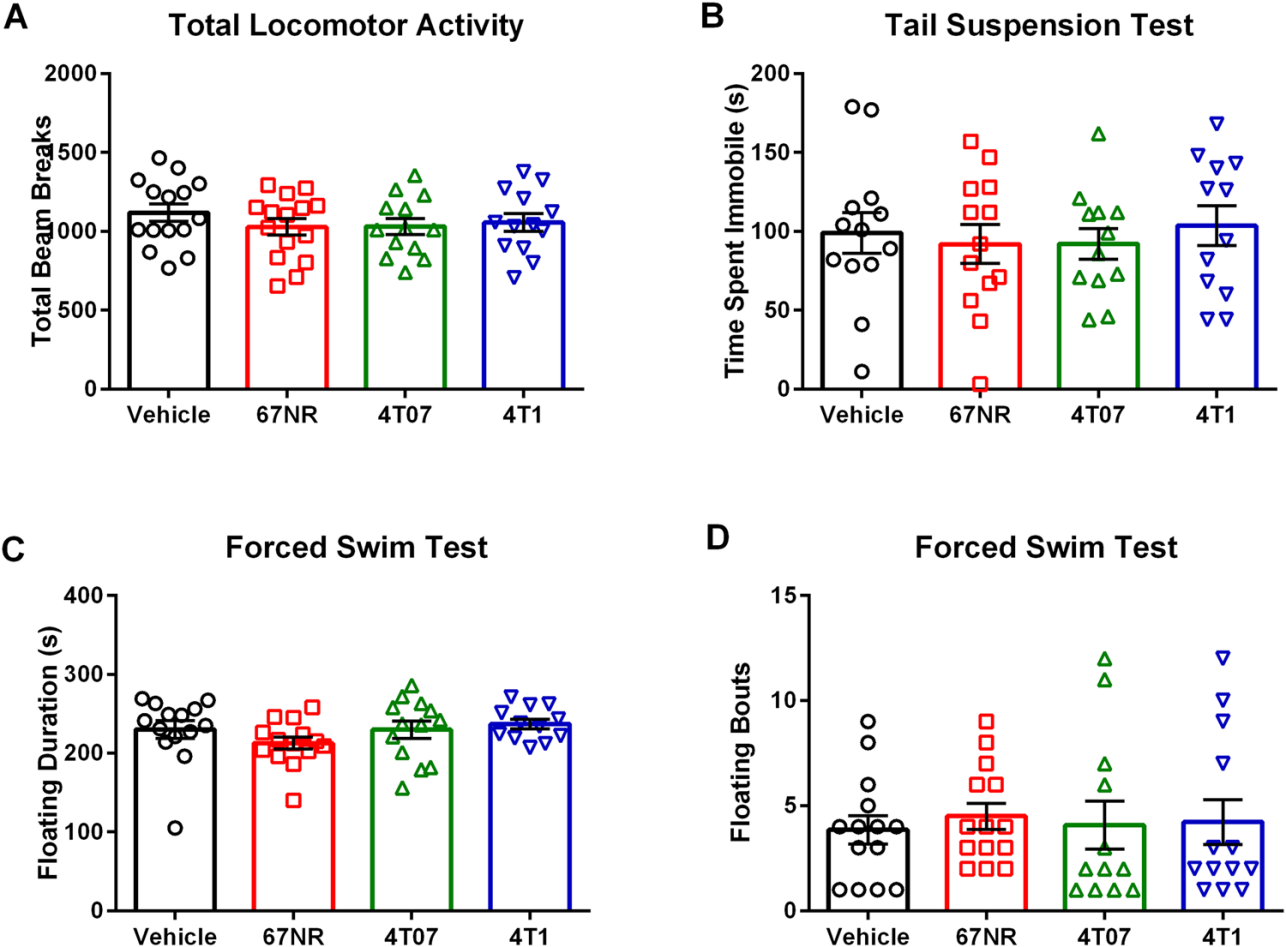
Locomotor Deficits or Depressive-like Behaviors are not Induced by Mammary Tumors. (**A**) Total number of beam breaks for vehicle and treatment groups during open field testing. There were no significant differences in total locomotor activity. (**B**) Immobility time during tail suspension test for vehicle and treatment groups. There were no significant differences in time spent immobile. (**C**–**D**) Average time spent floating and number of floating bouts for vehicle and treatment groups during forced swim test. There were no significant differences in floating duration or floating bouts. The measures and tissues were collected 20 days after tumor cell or vehicle inoculation (n = 12–15/group). The data are presented as mean + SEM. Graph bars that do not share a letter are statistically significant different at p < 0.05.

There were no group differences in total locomotor activity, anxiety-like behavior, or depressive-like behavior as measured by total beam breaks and central tendency during open field testing (Fig. 5A, F_3,52_ = 0.6453, p > 0.05; data not shown, F_3,51_ = 0.4920, p > 0.05), number of floating bouts, total time floating, and latency to float during the forced swim test (Fig. 5D, F_3,49_ = 0.0978, p > 0.05; Fig. 5C, F_3,49_ = 1.207, p > 0.05; data not shown, F_3,51_ = 0.7222, p > 0.05), and number of immobile bouts, time spent immobile, and latency to immobility during tail suspension (data not shown, F_3,46_ = 0.2459, p > 0.05; Fig. 5B, F_3,46_ = 0.222, p > 0.05; data not shown, H = 1.571, p > 0.05). To rule out a potential effect of timing of the behavioral tests, we tested a separate cohort of animals with the same treatment groups during the dark phase of the light-dark cycle. These mice also did not exhibit group differences in total locomotor activity or anxiety-like behavior as measured by total beam breaks and central tendency during open field testing (data not shown, H = 7.503, p > 0.05; data not shown, F_3,26_ = 1.148, p > 0.05), or depressive-like behavior as measured by number of floating bouts (data not shown, F_3,28_ = 0.5950, p > 0.05), and total time floating during the forced swim test (data not shown, F_3,27_ = 0.4108, p > 0.05).

### Cohort 2

#### Mammary Tumors Induce Central Pro-Inflammatory Cytokine Production but Have No Effect on Learned Fear or DCX + Cell Number

**Figure 6:**
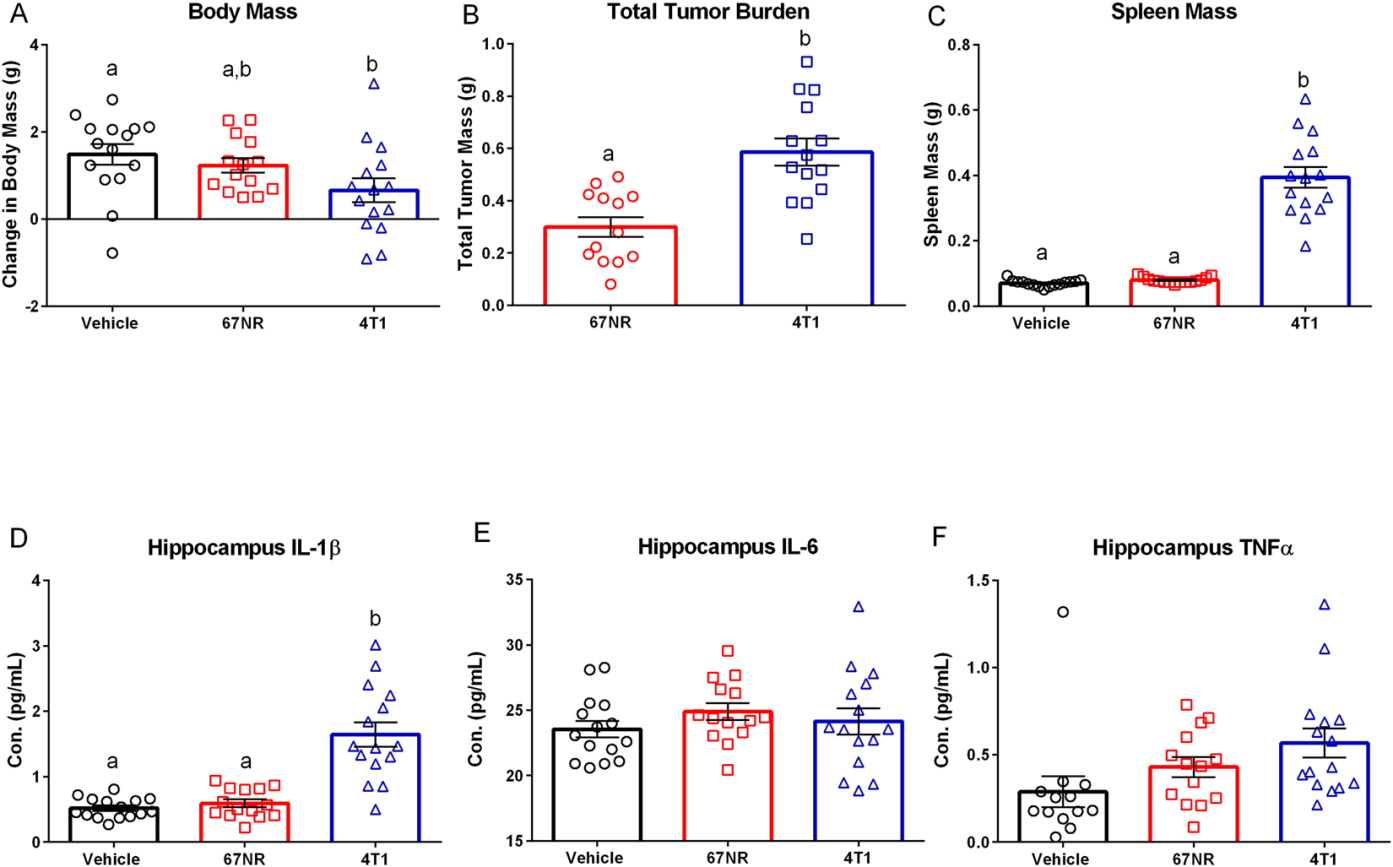
4T1 Cells Generate both a Peripheral and Central Response. (**A**) Body mass difference between day of tumor inoculation (DOI) and day of euthanasia (DOE) accounting for tumor mass (TTB) for vehicle and each cell line ((DOE-DOI)-TTB). 4T1 tumors had reduced weight gain relative to vehicles. (**B**) Total tumor burden in grams; 4T1 tumors had significantly increased mass. (**C**) Spleen mass for vehicle and treatment groups. Spleen mass was significantly increased in 4T1 tumors relative to 67NR tumors and vehicles. (**D**–**F**) Hippocampus protein cytokine concentrations of IL-1β, IL-6, and TNFα expressed in both vehicle and treatment groups. (**D**) Hippocampal IL-1β was significantly increased in 4T1 tumors relative to 67NR tumors and vehicles. (**E**) There were no significant differences in hippocampal IL-6. (**F**) TNFα was increased in the hippocampus of 4T1 treated mice relative to vehicles. The measures and tissues were collected 20 days after tumor cell or vehicle inoculation (n = 12–15/group). The data are presented as mean + SEM. Graph bars that do not share a letter are statistically significant different at p < 0.05.

**Figure 7:**
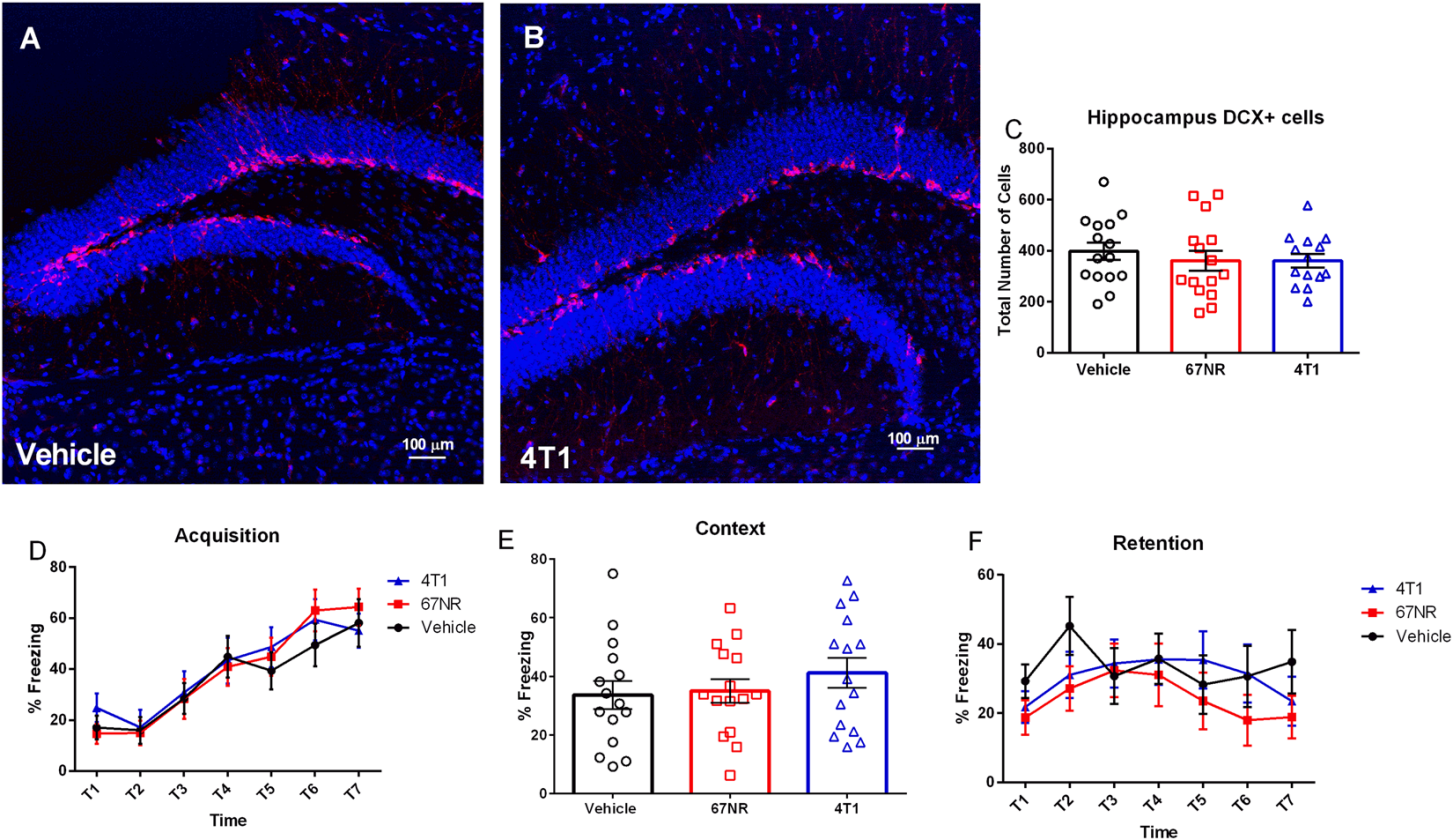
Learned Fear and Neurogenesis are not Altered by Mammary Tumors. (**A**,**B**) Representative IHC Staining of neurogenesis in the dentate gyrus of the hippocampus vehicle and 4T1 treatment groups (DCX in red and DAPI in blue). (**C**) Mean number of DCX + cells in the subgranular zone of the dentate gyrus. There were no significant differences in the number of DCX + cells. (**D**–**F**) Acquisition, context, and retention phases of fear conditioning testing. There were no significant differences in acquisition, context, and retention during fear conditioning. The measures and tissues were collected 18–20 days after tumor cell or vehicle inoculation (n = 12–15/group). The data are presented as mean + SEM. Graph bars that do not share a letter are statistically significant different at p < 0.05.

As reported in cohort one, 4T1 mammary tumors resulted in a significant reduction in body mass (Fig. 6A, F_2,41_ = 3.377, p < 0.05), increase in spleen mass (Fig. 6C, H = 31.48, p < 0.001), and increases in hippocampal cytokine concentrations of IL-1β (Fig. 6D, H = 24.59, p < 0.0001) compared to the non-tumor bearing vehicle-treated controls. In contrast to cohort one, total tumor mass was greater among the mice with 4T1 tumors relative to the 67NR tumors (Fig. 6B, p < 0.001, unpaired t-test) in this study; however, as reported for cohort one, mice with 4T1 tumors had significantly greater spleen mass (Fig. 6C, p < 0.05, Dunn’s multiple comparisons) and hippocampal IL-1β concentration (Fig. 6D, p < 0.05, Dunn’s multiple comparisons) than mice with 67NR tumors. There were no significant group differences in hippocampal concentration of IL-6 (Fig. 6E, F_2,41_, p > 0.005), TNFα (Fig. 6F, F_2,39_, p > 0.005) IFNγ (data not shown, F_2,40_ = 0.9132, p > 0.005), IL-10 (data not shown, F_2,41_ = 3.207, p > 0.005), CXCL1 (data not shown, F_2,41_ = 0.1753, p > 0.005), IL-4 (data not shown, F_2,40_ = 0.1969, p > 0.005), IL-5 (data not shown, F_2,40_ = 1.887, p > 0.005), IL-2 (data not shown, F_2,39_ = 0.08792, p > 0.005), or IL-12p70 (data not shown, F_2,41_ = 1.109, p > 0.005). Despite group differences in proinflammatory cytokine concentrations, there were no group differences in the number of doublecortin (DCX) positive cells (Fig. 7C, F_2,41_ = 0.3946, p > 0.05) or in the acquisition (Fig. 7D, F_2,42_ = 0.1625, p > 0.05), context (Fig. 7E, F_2,42_ = 0.7435, p > 0.05), or retention (Fig. 7F, F_2,42_ = 0.6356, p > 0.05) phases of fear conditioning.

## Discussion

This study establishes that despite similar tumor sizes there is significant variation among tumor cell lines in their ability to induce a peripheral and central immune response. Additionally, despite the acute central pro-inflammatory cytokine production in the 4T07 and 4T1 tumors there were no significant changes in DCX + cells, affective behavior, or learned fear. Tumors generated via all three cell lines had detectable concentrations of IL-1β, IL-6, TNFα, CXCL1 IFN-γ, IL-2, IL-4, IL-5, IL-10, and IL-12p70 (Fig. 1C). However, tumors generated with 4T07 or 4T1 cells had significantly greater IL-1β, IL-6, and TNFα concentrations when compared to tumors generated with 67NR cells (Fig. 1C). Additionally, mice with 4T07 or 4T1 tumors had increased IL-1β, IL-6, TNFα, IFNγ, IL-2, IL-10, IL-4, and IL-5 in the serum along with increased concentrations of IL-1β, IL-6, and TNFα in the liver relative to vehicles (Figs 2 and 3). A recent paper by Masri and colleagues^21^ reported that lung adenocarcinoma can alter liver metabolism, and this shift involves the STAT3-Socs3 inflammatory signaling axis. Considering mice injected with either 4T07 or 4T1 cells displayed increased IL-6 production and that IL-6 signals through the STAT3-Socs3 pathway; we examined mRNA expression of both liver STAT3 and Socs3. Indeed, 4T07 and 4T1 tumors resulted in increased STAT3 and Socs3 mRNA expression. Future studies should examine whether the association between liver inflammation and metabolism is specific to lung adenocarcinoma or exists in other types of cancer, such as breast cancer.

Importantly, tumors generated with 4T07 and 4T1 cells, but not 67NR cells, induced central pro-inflammatory cytokine production in both the hippocampus and cortex; specifically, there was a significant increase in the concentration of IL-1β for both 4T07 and 4T1 tumor bearing mice (Figs 4 and 6). Of note, there was no correlation between tumor or serum cytokine concentrations and concentrations of these cytokines in the brain, demonstrating that serum cytokine concentrations do not serve as an indicator of central pro-inflammatory cytokine production.

To determine whether the increases in these cytokines were functionally significant, mice underwent a series of behavioral tests. Previous studies examining the effects of increased IL-1β and TNFα in the brain have concluded that both are associated with increased depressive-like behavior and cognitive deficits^22–25^. However, in the current study, acute central pro-inflammatory cytokine production among the 4T07 and 4T1 tumor bearing mice was not associated with changes in depressive-like behavior. Mice displayed no changes in the number of floating bouts, total time floating, or latency to float during the forced swim test or the number of immobile bouts, time spent immobile, and latency to immobility during tail suspension test, both of which are well-characterized behavioral tests for depressive-like behavior (Fig. 5).

Cognitive dysfunction is another common complaint among breast cancer survivors. Tumor induced central pro-inflammatory cytokine production provides a potential mechanism through which cancer may impair cognition. Indeed, it is well documented that central pro-inflammatory cytokine production can alter neurogenesis through actions on neurotrophins, particularly decreases in concentrations of brain-derived neurotrophic factor (BDNF), and that reductions in neurogenesis have been associated with impaired cognitive performance^10, 26–29^. With this in mind, we examined DCX + cells (an approximate measure of neurogenesis) in the hippocampus along with mouse performance on fear conditioning, which is a test for learned fear that includes some cognitive performance measures which are dependent on the hippocampus and altered by central pro-inflammatory cytokine production^30–33^. In the current study, however, tumor-induced acute central pro-inflammatory cytokine production had no effect on fear conditioning performance. All groups were able to acquire the task (Fig. 7D), and there were no differences in either the context or retention phases of fear conditioning (Fig. 7E,F). Consistent with the behavioral data, there was an absence of a tumor effect on BDNF gene expression and DCX + cells (approximate measure of neurogenesis; Figs 4J and 7C). DCX staining was used in this study instead of the more specific BrdU/DCX/NeuN co-staining because it did not require daily handling and injection and eliminated possible contamination of the behavioral equipment through excreted BrdU.

Although two of the cell lines used in the current study resulted in acute central pro-inflammatory cytokine production, the affective and cognitive deficits reported previously to be associated with tumor-induced cytokine production were not apparent in the 4T1 tumor bearing mice. There are several experimental differences between the current study and previous studies that may have contributed to this discrepancy in affective and cognitive outcomes. Importantly, previous studies have used either carcinogens or nonorthotopic tumor models to examine the effects of tumor-induced central pro-inflammatory cytokine production on behavior^11–14, 34, 35^. Additionally, previous studies have differed in species tested^11, 34^,number of cells injected to initiate the tumor, size of tumors, and timing of tissue collection^12–14, 35^.

Clinical studies have demonstrated an increased incidence of depression and cognitive deficits in cancer patients prior to any treatment^36, 37^. Of note, incidence rates of depression and cognitive deficits increase during cancer treatment^1, 37^. However, this study sought to examine the behavioral effects of peripheral tumors prior to any medical intervention. In the current study, sizeable mammary tumors did not induce behavioral deficits in Balb/C mice within our timeline. Time is a limiting factor in these studies due to the exponential growth pattern of the tumors. Indeed, the potential for confounding effects of tumor size on behavior increases as the tumors grow and alter locomotor activity. For the current study, a time point was chosen for behavioral testing that coincided with increased CNS cytokine expression in prior tumor cell line studies^13, 38^ but prior to the onset of locomotor deficits. Although there are examples of behavior deficits emerging within hours of increased CNS cytokine expression^39, 40^, it is possible that longer-term exposure to increased tumor-associated pro-inflammatory cytokine concentrations would have led to behavioral deficits. Future studies conducting a time course with slower growing tumors may be able to address this question. Interestingly, cognitive and affective disorders also can emerge in response to tumor induced sleep disruption; sleep disturbances are reported in 30–50% of cancer patients^41^. Central pro-inflammatory cytokine production can contribute to sleep alterations that persist over time, in turn eliciting affective changes and cognitive deficits^42–45^. Future directions should compare these cells lines’ effects on sleep. If reproduced in mouse models of cancer, then sleep deficits could have the potential to serve as predictors of future behavioral deficits, ultimately allowing for earlier interventions and prevention.

The mice in the current study were individually housed because social interactions can influence tumor growth^46, 47^ and CNS cytokine expression^48, 49^, which in turn would make data collected from the two animals within the cage non-independent. Although individual housing may have affected baseline behavior^50^, the approach is well-founded given that socially isolated cancer patients are more susceptible to affective disorders^51, 52^. Furthermore, social isolation typically amplifies CNS cytokine expression in the context of an inflammatory stimulus and the associated behaviors^49^. Regardless, the role of social interactions in the development of physiological and behavioral sequelae of cancer is an intriguing question that warrants examination.

The current study provides several examples of cell-line specific physiological changes despite similarly sized tumors, but the underlying causes remain unknown. Interestingly, the two cell lines that were associated with increased serum, liver, and brain inflammation in the current study have the ability to undergo epithelial to mesenchymal transition and leave the primary site (4T07 and 4T1), whereas the non-metastatic cell line (67NR) was not associated with increased cytokine concentrations in serum, liver or brain. Indeed, 4T1 tumors may result in lung and lymph node micro-metastasis as early as 14 days post-injection^53^, but this is unlikely to be the full explanation because there were very few significant differences in inflammatory outcome measures between the 4T1 and 4T07 groups even though the 4T07 group would not be expected to have metastases^20^. Additional studies are needed to determine the specific characteristics of these mammary tumors that are responsible for the exaggerated proinflammatory response observed in multiple organ systems.

In sum, 4T07 and 4T1 mammary tumors have the ability to induce peripheral and central immune responses. However, despite the acute central pro-inflammatory cytokine production produced by the 4T07 and 4T1 tumors, there were no significant changes in DCX + cells (an approximate measure of neurogenesis), affective behavior, or learned fear relative to vehicle treated control mice and those with 67NR tumors. These data demonstrate that the physiological and behavioral effects of mammary tumors are cell-line specific and that short-term central pro-inflammatory cytokine production does not produce behavioral deficits. Furthermore, these data imply that the cause of cognitive and affective changes among breast cancer survivors are more likely due to the effects of cancer treatments, than the tumor, on brain tissue. Further study of the independent and interactive effects of tumors and pharmacological treatments for cancer on the brain are warranted.

## Methods

### Animals

Adult (>7 weeks) female Balb/C mice were obtained from Charles River Laboratories. Mice were individually housed and allowed *ad libitum* access to food and filtered tap water throughout the course of the experiment. Cohort one consisted of 60 mice which were randomly assigned groups consisting of the vehicle (DMEM) control treatment or tumors generated using one of three tumor cell lines (n = 15 vehicle, n = 15 67NR, n = 15 4T07, n = 15 4T1). The mice received 100 μl orthotopic bilateral injections of either tumor cells (1 × 10^5^ per injection; cell lines described in detail below) or vehicle (DMEM) into the fourth and ninth inguinal mammary glands. On the morning of Day 20 following tumor inoculations, mice underwent behavioral testing which consisted of open field (assesses locomotor behavior and anxiety-like behavior), tail suspension (assesses depressive-like behavior), and forced swim testing (assesses depressive-like behavior). Approximately 4 h after testing, body mass was recorded, a blood sample was collected from the submandibular vein, and then the mouse was euthanized for tissue collection; spleen, liver, and tumors were dissected and placed in sterile tubes containing RNA later (Invitrogen, Waltham, MA). The brains were dissected into the left and right hemispheres and placed in tubes containing RNA later (Invitrogen, Waltham, MA) and stored at −80 °C; two days later, the cortex and hippocampus were dissected from both hemispheres, one hemisphere for processing for PCR (gene expression) and the other hemisphere for analysis of protein cytokine levels via multiplex assay. To determine whether any groups experienced significant weight loss, body mass measurements were obtained for each mouse and subtracted from individual body mass on the day of injection. Next, total tumor burden was subtracted from this difference to account for the mass of the tumor ((Body Mass Day of Euthanasia- Body Mass Day of Injection)- Total Tumor Burden). Cohort two consisted of 45 mice (n = 15 vehicle, n = 15 67NR, and n = 15 4T1) that were randomly assigned to experimental groups. All mice received 100 μl orthotopic bilateral injections of either tumor cells (67NR or 4T1, 1 × 10^5^ per injection) or vehicle (DMEM) into to the fourth and ninth inguinal mammary glands. Mice underwent learned fear assessment via fear conditioning; the acquisition training occurred during the light period of Day 18 after tumor induction and then context training (light period) and retention testing (dark period) occurred the following day. On Day 20, body mass was recorded, a blood sample was collected from the submandibular vein, and then the mouse was euthanized for tissue collection; spleen, liver, and tumors were dissected and placed in sterile tubes containing RNA later (Invitrogen, Waltham, MA). The brains were split into the left and right hemispheres; one hemisphere was placed in RNA later (Invitrogen, Waltham, MA) and processed for the multiplex assay as described above, while the other hemisphere was post-fixed in 4% paraformaldehyde overnight, and then transferred to a 30% sucrose solution. After cryoprotection, brains were frozen at −80^o^ C until sectioning. All experiments were approved and carried out in accordance with guidelines set by The Ohio State University Institutional Animal Care and Use Committee.

### Cell Lines and Orthotopic Injections

67NR, 4T07, and 4T1 cells were obtained from Barbara Ann Karmanos Cancer Institute (Detroit, MI). These cell lines are distinct isolated subpopulations from a single spontaneous Balb/cfC3H mammary tumor, and differ in their ability to induce secondary metastasis. 67NR cells fail to leave the primary site, while 4T07 cells have the ability to leave the primary site but fail to complete the metastatic cascade and form visible metastases. However, 4T1 cells both leave the primary site and form visible secondary metastases^20^. Cells were cultured in high glucose Dulbecco’s Modified Eagle Medium (DMEM) with L-glutamine (Gibco11965) supplemented with 10% certified heat inactivated fetal bovine serum (Gibco 10082), 1% antibiotic-antimycotic (Gibco 15240), and 0.12% mixed nonessential amino acids (Gibco 11140). Cells were maintained in an incubator at 37 °C with a mixture of 5% carbon dioxide and 95% air. Prior to injections cells underwent mycoplasma testing using the PlasmoTest kit (InvivoGen, San Diego, CA) and were verified to be free of any mycoplasma contamination. Cell passage numbers were the same throughout the experiment. Cells were diluted in a 1:1 mixture of trypan blue (Life Technologies, Carlsbad, CA) and were counted using an automated cell counter (Cell Countess II, Life Technologies, Carlsbad, CA). For injections cells were diluted in DMEM (Gibco 11965) for a concentration of 1 × 10^5^ per 100 μl. Mice were briefly anesthetized using isoflurane and a small 2–3 mm midline incision was made directly between the fourth and ninth inguinal mammary glands, allowing visualization of both glands. Each mammary gland was injected with 100 μl of either tumor cells (1 × 10^5^ cells) or vehicle (DMEM). As tumors became palpable, measurements were obtained every three days using sliding calipers; tumor volume was calculated using the following formula: tumor volume = (length × width^2^)/2^54^. Mice displaying skin ulceration or tumor size meeting the endpoint criteria (1.5 cm in any direction) prior to conclusion of the study were euthanized immediately and not included in data analyses (n = 4 in cohort 1).

### RNA Extraction, cDNA, and PCR

RNA was extracted using Trizol Reagent (Ambion, Waltham, MA) according to the manufacturer’s instructions. RNA quantity and quality was determined using a spectrophotometer (Nanodrop One, Wilmington, DE). cDNA was synthesized using M-MLV reverse transcriptase and diluted 1:10 for subsequent PCR. Inventoried probes from Applied Biosystems (Life Technologies, Carlsbad, CA) were used (Table 1). Taqman Fast Advanced Master Mix (Life Technologies, Carlsbad, CA) containing AmpliTaq Fast DNA Polymerase were used in a 20 μL duplex reaction with one probe (Table 1) and a primer-limited probe for the endogenous eukaryotic control 18 s rRNA. The 2-step real-time PCR cycling conditions used were 95 °C for 20 s, 40 cycles of 95 °C for 3 s, and then 60 °C for 30 s. Gene expression was quantified using the Pfaffl Method^55^.

**Table 1:** Gene names and assay ids for TaqMan primer/probe sets used throughout the experiment.

Gene Name	Assay ID
*IL-1β*	Mm00434228_m1
*IL-6*	Mm00446190_m1
*TNFα*	Mm00443258_m1
*Socs3*	Mm00545913_s1
*Stat3*	Mm01219775_m1
*NFKb1*	Mm00476361_m1
*IFNγ*	Mm01168134_m1
*Ido1*	Mm01218006_m1
*BDNF*	Mm01334044_m1
*Itgam (CD11b)*	Mm00434455_m1
*Nos2 (iNos)*	Mm00440502_m1
*Dlg4 (PSD-95)*	Mm00492193_m1
*IDO2*	Mm00524210_m1
*18s*	Hs99999901_s1

### Protein Multiplex

Prior to running the multiplex, extracted hippocampi, cortex, and tumors were first sonicated in RIPA buffer and allowed to sit on ice for 30 min. Following 30 min incubation samples were then centrifuged at 13,300 rpm for 15 min at 4 °C and supernatant was taken. Pierce BCA Protein Assay Kit was then run to insure equal amount of protein was loaded into multiplex. To determine cytokine protein levels serum, tumor, cortex, and hippocampus samples were analyzed using Meso Scale Discover V-Plex Pro-inflammatory Mouse Panel according to manufacturer’s instructions. The kit measures protein levels of the following cytokine and chemokines: IFNγ, IL-10, IL-12p70, IL-1β, IL-2, IL-4, IL-5, IL-6, KC/Gro (CXCL1), and TNFα. The plates were run on a Meso Quickplex machine and the data analyzed using MSD Discovery Workbench software v4.0.

### DCX Staining and Quantification

The paraformaldehyde and sucrose treated brains from Cohort 2 were sectioned at 30 μm into four series using a cryostat. Only series one was used for the doublecortin (DCX) staining. Free-floating brain sections were washed three times with PBS (pH 7.4; 5 min each), and then treated with Tris-EDTA (pH 8.0; 10 min) for antigen retrieval. After three additional washes with PBS (5 min each), sections were treated with 2.5% normal horse serum dissolved in 10 m*M* PBS containing 0.1% Triton-X-100 (PBSHT) for 45 min to block non-specific binding. Next, sections were incubated with a goat polyclonal anti-DCX antibody (dilution 1:200; sc-8066; Santa Cruz Biotechnology) in PBSHT overnight at room temperature. Thereafter, sections were washed three times in PBS (5 min each) and incubated with the donkey anti-goat immunoglobulin G conjugated to Alexa Fluor 594 (dilution 1:200; A11058; Invitrogen) in PBSHT for 2 h at room temperature. Finally, sections were washed three times in PBS for 5 min each, mounted onto glass slides, treated with Vectashield mounting medium with DAPI (H-1500; Vector Laboratories Inc.), coverslipped and visualized in the Cy3 channel (Zeiss AxioImager M2). All images of the subgranular zone of the dentate gyrus were taken at 20x magnification. All DCX + cells in the subgranular zone were counted by an experimenter blind to experimental assignments.

### Behavioral Testing

#### Open Field Testing

Mice were placed for 10 min in a polypropylene open field arena (36 cm × 36 cm) with two rows of infrared sensors mounted on the sides of the box to detect movement (Open Field Photobeam Activity System, San Diego Instruments Inc). These boxes are contained in cabinets which allow for experimenter manipulation of lights and attenuation of outside sound. For each mouse total activity (i.e. beam breaks) and central tendency was calculated and analyzed.

#### Forced Swim Test

Mice were placed for 5 min in a 5000 ml beaker filled with 3500 ml of water at ~27 °C. Mice were videotaped and their behavior was later scored using The Observer XT 8.0 software (Noldus, Leesburg, VA). Latency to float, time spent floating, and number of floating bouts were calculated and analyzed for each mouse.

#### Tail Suspension Test

Mice were suspended for 5 min by their tail, which was taped to the top edge of a 92 cm high box. Mice were videotaped and their behavior was later scored by hand using The Observer XT 8.0 software (Noldus, Leesburg, VA). Time spent immobile was calculated and analyzed for each mouse.

#### Fear Conditioning

This test is comprised of three testing sessions (acquisition, context, retention) over two days. The testing apparatus (MedAssociates, St. Albans, VT) consists of a Plexiglas box which contains a metal floor through which a shock is administered. The entire Plexiglas box is housed inside a cabinet that blocks out both external light and sound. A video camera is mounted on the door of the chamber and records the movement and freezing of each mouse. Time spent freezing for each mouse was calculated and analyzed using VideoFreeze software (MedAssociates, St. Albans, VT). Acquisition trial (Day 18) consisted first of a 180 sec acclimation period, followed by an 80 dB tone for 20 sec and a 0.6 mA shock during the last second of the tone. This 20 sec tone and shock pairing was administered seven times with a 30 sec inter-trial interval. This trail concluded with a 60 second observation period. Following the trial mice were placed back in their home cage. The context trial (Day 19 morning) consisted of each mouse being placed in the Plexiglas box for 180 seconds with no tone or shock administered. Behavior was recorded and following completion of the trail the mice were returned to their home cages. The retention trial consisted first of changing the environment to make it less recognizable for the mice. White inserts were used both to cover the metal grid floor and change the shape of the box to semi-circular. Vanilla extract was placed in the bottom of the box to change the odor. The trial design was exactly the same as the acquisition trail except no shock was administered. Following completion of the trial, mice were returned to their home cages.

### Statistical Analysis

Outliers were identified and subsequently removed prior to analyses using the Grubb’s Test. All data except for retention and acquisition stages of fear conditioning were analyzed using a one-way ANOVA. Post-hoc analyses were performed using Tukey’s multiple comparisons test. If data did not meet the assumptions of ANOVA, then statistical analyses were conducted using a nonparametric Kruskal-Wallis test. Post-hoc analyses on nonparametric data were performed using Dunn’s multiple comparisons test. For both the acquisition and retention stages of fear conditioning two-way repeated measures ANOVA was used for subsequent analyses. Post-hoc analyses were performed using Tukey’s multiple comparisons test. Correlations were assessed using Pearson’s correlations. P < 0.05 was considered significant for all data except those acquired using the Meso Scale Discover V-Plex Pro-inflammatory Mouse Panel. To account for the simultaneous measurement of 10 analytes in the same sample p < 0.005 was considered significant (0.05/10 = 0.005). All statistical analyses were performed using GraphPad Prism 6.0 software.

### Data Availability

The data that support the findings of this study are available (in raw form) from the corresponding author upon reasonable request.
